# Research Progress and Treatment Status of Liver Cirrhosis with Hypoproteinemia

**DOI:** 10.1155/2022/2245491

**Published:** 2022-02-24

**Authors:** Jianxia Wen, Xing Chen, Shizhang Wei, Xiao Ma, Yanling Zhao

**Affiliations:** ^1^School of Food and Bioengineering, Xihua University, Chengdu 610039, China; ^2^Department of Pharmacy, Chinese PLA General Hospital, Beijing 100039, China; ^3^Pharmacy Department, Chongqing Emergency Medical Center, Chongqing University Central Hospital, Chongqing 400014, China; ^4^College of Pharmacy, Chengdu University of Traditional Chinese Medicine, Chengdu 611137, China

## Abstract

Liver cirrhosis is the 14th leading cause of death in adults worldwide. The liver is an important organ for the metabolism of sugar, protein, and fat. Liver cirrhosis with hypoproteinemia (LCH) can lead to metabolic disorders of the nutrients such as sugar, protein, and fat, as well as insufficient protein intake, digestion and absorption disorders, and continuous leakage of plasma protein into the abdominal cavity. Severe hypoproteinemia leads to a poor prognosis in patients. For every 10 g/L decrease in peripheral blood albumin, the risk of secondary liver disease complications will increase by 89% and the mortality rate increased by 24%–56%. Therefore, it is necessary to take urgent measures to treat liver cirrhosis with hypoalbuminemia and effectively treat and reverse the deterioration of the disease caused by hypoalbuminemia, so as to reduce the burden of secondary liver disease. Emerging evidence suggests that protein balance disorders, auxin resistance, and hyperleptinemia are key steps in the development of cirrhosis and hypoproteinemia. This study comprehensively analyzed the common complications, pathogenic mechanisms, and treatment status of cirrhosis caused by hypoproteinemia and proposed research prospects for dealing with this increasingly serious problem.

## 1. Introduction

Liver cirrhosis with hypoproteinemia (LCH) is a common clinical manifestation of liver cirrhosis, which is a chronic liver disease caused by continuous and repeated actions on the liver by a single or a complex cause. In patients with liver cirrhosis, due to the decline of liver cell function and the decrease in the number of liver cells, the protein synthesis including albumin and various coagulation factors is insufficient, resulting in hypoproteinemia [1]. A substantial reduction in the content of albumin in the body will accelerate the progression of liver insufficiency, decrease immunity, and increase the chance of infection, ascites, and hypovolemia, reduce organ dysfunction, and so forth [2], which seriously affects patient prognosis and long-term survival, and the fatality rate is high. Studies have shown that a reduction in serum albumin could increase the mortality of patients. For every 2.5 g/L decrease in peripheral albumin, the risk of secondary liver disease complications increased by 89% and the mortality rate increases by 24%–56% [3].

Currently, the improvement of hepatic protein synthesis function and hypoalbuminemia through drug therapy has become the focus of many hepatologists. However, clinical treatment faces problems such as shortage of blood product resources, high price, blood disease infection, and blood transfusion reaction. Therefore, the treatment of LCH still has certain limitations, and other alternative treatments are urgently needed. The research progress on LCH at home and abroad is summarized as follows.

## 2. Methods

The databases, including PubMed, SINOMED, Web of Science, EMBASE, China National Knowledge Infrastructure (CNKI), Wanfang, Chinese Biomedical Database (CBM), and VIP medicine information system (VMIS), were comprehensively searched. The following search terms were used: “liver cirrhosis” [Mesh terms] OR “hypoproteinemia” [Mesh terms]. Studies concerning the research progress and treatment status of cirrhotic hypoproteinemia were picked out manually. Two investigators independently reviewed studies after reading the title, abstract, and full text of the related studies. Any relevant disagreements were resolved by consultation with Professor Zhao.

## 3. Complications of LCH

### 3.1. Gastrointestinal Bleeding

Gastrointestinal bleeding is closely associated with the complicated course of LCH, which reflects the severity of bleeding events [4, 5]. Patients with liver cirrhosis are usually accompanied by portal hypertension, gastric ulcer, gastric lesions, and other diseases. Esophageal and gastric fundus veins are prone to rupture in the varices and cause gastrointestinal bleeding. In addition, gastric mucosa is also prone to erosive bleeding, and patients with LCH complicated with gastrointestinal bleeding have a higher mortality rate [6, 7].

### 3.2. Spontaneous Peritonitis

Patients with cirrhosis and hypoalbuminemia often have ascites or edema when the plasma albumin concentration is lower than 30 g/L, and ascites is bound to occur when it is lower than 25 g/L. Due to the impaired liver function, the body's defense function is low, intestinal bacteria multiply and translocate, and bacterial infection is complicated [8, 9]. Spontaneous peritonitis (SBP) is the most common complication of liver cirrhosis. The onset of SBP is relatively insidious, early diagnosis is difficult, and the end-stage cure rate is low. Therefore, it is also the leading cause of hospitalization and death in advanced patients [10].

### 3.3. Hepatorenal Syndrome

Hepatorenal syndrome is a serious complication of LCH. It is a syndrome characterized by renal failure caused by liver dysfunction, decreased renal blood perfusion, and abnormal endogenous vascular activity. It is associated with many factors such as secondary infections, liver damage caused by drugs, varicose vein rupture, and bleeding. The incidence of hepatorenal syndrome in LCH is 18% and 40% in 1 and 5 years, respectively [11]. Once it occurs, the prognosis is extremely poor. Studies [12] have shown that the mortality rate of hepatorenal syndrome in cirrhosis is as high as 52% to 90%, and the mortality rate can reach 100% without inappropriate interventions.

## 4. Pathogenesis of LCH

### 4.1. Impaired Protein Balance

Patients with liver cirrhosis often have symptoms of gastrointestinal dysfunction such as abdominal distension, loss of appetite, nausea, and vomiting, as well as gastrointestinal mucosal diseases such as portal hypertensive gastropathy, small bowel disease, and enteropathy, resulting in decreased protein intake and protein absorption. In liver cirrhosis, the original liver lobules are destroyed, forming false lobules, and the sinusoidal channels are disordered, which reduce the nutrient supply and oxygen supply of liver cells, aggravate the necrosis of liver cells, and reduce the synthesis of blood albumin by more than 50% [13, 14]. Studies have shown [15] that patients with liver cirrhosis are in a state of high metabolism and high decomposition, and their protein metabolism and decomposition are significantly faster than those of normal people, and the protein decomposition speed is faster than the synthesis rate. In addition, the liver glycogen content in patients with liver cirrhosis is only 1/2 of that of the normal liver, and there are obstacles to glucose utilization. Plasma insulin, glucagon, and epinephrine levels increase, which promotes early protein gluconeogenesis to maintain the body. There is a need to further reduce the serum albumin level in humans. In addition, complications such as ascites, peritonitis, and gastrointestinal bleeding can lead to substantial protein loss. Therefore, a variety of factors lead to impaired protein balance in patients with liver cirrhosis, which induces LCH.

### 4.2. Growth Hormone Resistance

Growth hormone (GH) is the main hormone that promotes protein synthesis in the body. In recent years, domestic and foreign scholars have studied the growth hormone-insulin-like growth factor (IGF-Ι) axis and found that it regulates the energy metabolism of glucose, protein, and fat. GH, which plays an important role, is mostly mediated by IGF-I, while the synthetic release of IGF-I is caused by GH stimulation, and IGF-I is mainly synthesized and released by hepatocytes. There is growth hormone resistance in patients with liver cirrhosis, which leads to obstacles in the synthesis of IGF-I and its binding protein in the liver, resulting in decreased liver albumin synthesis and decreased glucose gluconeogenesis and glycogen synthesis. It is currently believed that insulin resistance and high peripheral blood growth hormone levels play an important role in malnutrition due to liver cirrhosis [2].

### 4.3. Hyperleptinemia

Leptin is a protein product of obesity-related genes. It is mainly produced and secreted in adipose tissue. It senses the energy reserves of fat cells and is an important regulator of the hypothalamic axis involved in the regulation of food intake. In recent years, a number of studies have shown that hyperleptinemia is closely related to the occurrence and development of hypoalbuminemia cirrhosis [16]. Wang et al. [17] indicated that serum leptin levels in patients with hypoalbuminemia in liver cirrhosis are significantly increased, and peripheral leptin receptors can promote low protein levels by suppressing appetite, promoting fat consumption and carbohydrate conversion, and reducing body weight and the development of blood diseases. Campillo et al. [18] found that blood leptin levels are positively correlated with related indicators of hypoalbuminemia, indicating that hyperleptinemia plays an important role in cirrhotic malnutrition, and to a certain extent can be used as an effective diagnosis of hypoalbuminemia in liver cirrhosis index. In addition, Feng et al. [19] explored the relationship between leptin, liver cirrhosis, and hepatocellular carcinoma (HCC), confirming that leptin is an independent risk factor for the progression of hepatitis B cirrhosis to liver cancer and is involved in evaluating the body's nutritional level. Hepatitis B cirrhosis is a potential serum factor for HCC.

## 5. Treatment of LCH

### 5.1. Modern Medical Treatment of LCH

#### 5.1.1. Antivirus

Antiviral therapy is the most routine intervention in the treatment of LCH in modern medicine [20]. Antiviral drugs can reduce hepatitis B (HBV) replication, prevent further necrosis of liver cells, improve liver function to varying degrees, promote the recovery of liver synthesis function, and increase serum albumin concentration. Shang [21] reported the application of lamivudine combined with conventional liver protection measures to treat 93 patients with cirrhosis and hypoalbuminemia, and serum albumin increased significantly after 6 months of treatment. Lamivudine can significantly improve the liver function of patients with liver cirrhosis and increase the level of serum albumin. Another group of clinical controlled studies have shown that entecavir has strong anti-hepatitis B virus activity and is well tolerated [22]. It can effectively inhibit HBV replication, reduce hepatocyte inflammatory response, and improve vascular endothelial cell function. The treatment effect for hypoproteinemia, abdominal effusion, and portal hypertension is remarkable.

#### 5.1.2. Human Albumin or Plasma Infusion

Infusion of human albumin or plasma is currently one of the most commonly used methods for the treatment of hypoalbuminemia in liver cirrhosis. A number of studies have clearly shown that albumin can effectively improve hemodynamic disorders and various complications caused by advanced liver cirrhosis. All exhibited this common characteristic. Salerno et al. [23] confirmed the efficacy of albumin in improving the outcome of SBP patients through meta-analysis and significantly improved the survival rate of hospitalized patients. When liver cirrhosis is complicated by HRS, albumin combined with vasoconstrictor (mainly Terlipressin) can improve the survival of patients in a short period of time. It can improve renal function in about 40% of cases and cure HRS in about 30% of cases [24]. Bernardi et al. [25] analyzed 17 trials and found that albumin infusion was more effective in reducing circulatory dysfunction after paracentesis and reducing the incidence and mortality of ascites compared with conventional therapy.

However, due to the lack of clear clinical data and clinical efficiency evaluation, the role of albumin in some complications of liver cirrhosis has been controversial. For example, there is little evidence on the effect of albumin fold treatment in patients with non-SBP-related infections. A recent randomized study showed that treatment with albumin in combination with antibiotic improved the circulation and renal function in patients, but it was not observed between the two groups. It has a significant effect on the incidence of renal failure, and there is no difference in the 3-month cumulative survival rate. Therefore, further studies are needed to elucidate the therapeutic effect of albumin under this condition [26]. Although clinical studies have shown that transfusion of human albumin is beneficial in patients with severe HE, it is unclear whether cirrhosis complicated by hepatic encephalopathy is an indication for albumin due to the lack of clear clinical efficacy evaluation.

#### 5.1.3. Amino Acid Supplementation

When hypoalbuminemia occurs in liver cirrhosis, the metabolism of amino acids and proteins in the body is mainly manifested as increased blood ammonia, decreased plasma albumin, and changes in the amino acid profile in the blood. Studies have shown that, in the amino acid metabolism disorder in the early stage of liver disease, the main reason is the reduction of branched chain amino acids (BCAA). Branched chain amino acids are essential raw materials for human protein synthesis and can be used as applied substances to provide the body with the necessary energy supply and reduce protein breakdown [27]. Clinically, compound amino acid injection has a wide variety of uses due to different uses. Amino acid injection has a high proportion of branched chain amino acids, which can help correct the imbalance of various amino acid ratios in patients with liver cirrhosis. It is often used for nutritional support treatment of patients with liver cirrhosis. Clinical studies have shown that the serum albumin concentration of patients with liver cirrhosis increases significantly after using branched chain amino acids, the incidence of ascites decreases, and the Child-Pugh score is reduced [28]. Ohno et al. confirmed through long-term clinical trials that supplemental therapy with branched chain amino acids can increase serum albumin levels in patients with liver cirrhosis [29] and reduce the risk of HCC and other complications [30].

#### 5.1.4. Recombinant Human Growth Hormone

Growth hormone is a hormone secreted by the anterior pituitary gland, which can promote liver cell regeneration and albumin synthesis. It is difficult to obtain natural growth hormone. Artificially synthesized recombinant human growth hormone is commonly used in clinical practice, which has exactly the same structure and function as natural growth hormone. Recombinant human growth hormone can reverse the negative nitrogen balance and has an obvious “nitrogen storage” effect, thereby stimulating the expression of liver cell protein mRNA and increasing the body's protein level. Studies have shown [30, 31] that patients with liver cirrhosis have severe growth hormone resistance, and their serum GH levels are elevated, leading to reduction of the levels of IGF-1 and insulin-like growth factor binding protein-3 (IGFBP-3). Exogenous recombinant human growth hormone can overcome growth hormone resistance and increase the level of IGF-1 and IGFBP-3. IGF-1 can inhibit protein breakdown and increase amino acid intake and cell proliferation, and IGFBP-3 can upregulate the biological activity of IGF-1 and further promote anabolism. Luo et al. [32] explored the effect of recombinant human growth hormone (rhGH) on the residual liver after hepatectomy in 24 patients with cancer and cirrhosis. The results showed that hepatic functional damage immediately after hepatectomy is a significant risk factor for early intrahepatic recurrence in cirrhotic HCC. The rhGH can promote the recovery of liver function by reducing the decline of cellular immune function in patients with liver cancer and liver cirrhosis and can also promote the body's protein synthesis and liver regeneration.

### 5.2. Traditional Medicine Treatment for LCH

#### 5.2.1. Fubai Formula

Traditional Chinese medicine (TCM) believes that albumin belongs to the subtle category of qi and blood, while the spleen is the official source of qi and blood. Modern medicine shows that the drugs in Fubai prescription can not only directly supplement albumin but also reduce liver cell degeneration and necrosis, improve liver microcirculation, inhibit fibrous tissue proliferation, and significantly increase the infiltration of 3H-leucine into serum and liver to participate in protein synthesis, thereby improving the albumin level [33]. Shi et al. [34] analyzed the syndrome characteristics of patients with liver cirrhosis and hypoalbuminemia and the treatment effect of TCM, and the results showed that Fubai formula was significantly better than the control group in terms of TCM symptom improvement and serum albumin normalization rate. The long-term effect was more significant and no adverse reactions were seen. Serum cholinesterase (ChE) can sensitively and specifically reflect the anabolic function of the liver, and it is a sensitive indicator for evaluating the protein synthesis function of liver cells [35]. Xue [36] explored the effect of Fubai prescription on serum cholinesterase activity in patients with hepatitis and liver cirrhosis and found that the efficacy of Fubai prescription was significantly better than that of the control group. This is mainly related to the complex of Fubai Fang in promoting hepatocyte regeneration and eliminating immunity and increasing the activity of serum ChE.

#### 5.2.2. Wuji Baifeng Pills

The prescription contains 30% black-bone chicken and is matched with Ginseng Radix et Rhizoma and Astragali Radix to invigorate *qi* and promote fluid. It is supplemented with Chuanxiong Rhizoma, Angelicae Sinensis Radix, Paeoniae Radix Alba, and Rehmanniae Radix Praeparata to nourish *qi* and blood, regulate menstruation, and relieve pain, and Cyperi Rhizoma, Dioscoreae Rhizoma, and Cervi Cornus Colla are added to regulate *qi* and invigorate the spleen and antler gum, as well as nourishing blood and *yang*. Hu et al. [37] showed that Wuji Baifeng Pill can protect liver cells from damage and regulate amino acid imbalance. It is also conducive to the synthesis of albumin and forms a virtuous circle. The study by Zhan et al. [38] and other studies have shown that the Wuji Baifeng Pill treatment group can improve the clinical symptoms, liver function, and blood coagulation function of patients more effectively than the control group and increase the serum total protein and albumin content of the patient. The total effective rate of the treatment group is 83.3%, was significantly higher than that of the control group (53.1%), and there were no adverse reactions during treatment. Therefore, Wuji Baifeng Pill is an ideal drug prescription for treating LCH. The main prescriptions of the TCM are listed in Table 1.

#### 5.2.3. Shengbai Decoction

Ye and Wu [42] used liver hydrolyzed peptide intravenous infusion on the basis of routine liver protection, enzyme reduction, support, and diuresis, and the treatment group received Shengbai decoction with significantly better therapeutic effect than the control group. The treatment group was better than the control group in terms of restoring biochemical indicators, inhibiting HBV-DNA and HBeAg negative, and improving Child-Pugh score was also significantly higher than the control group. Zhu et al. [39] have shown that Shengbai decoction is better than the control group in improving the clinical symptoms of patients and improving the survival rate of patients. This may be related to the reduction of GH and LEP levels, improvement of insulin resistance, and adjustment of IGF-Ι and GH. The negative feedback is related to the increased albumin level.

#### 5.2.4. Qijia Chaizhu Decoction

Kong et al. [40, 41] compared the efficacies of Qijia Chaizhu decoction and conventional liver-protecting therapy in 60 patients with clinical liver cirrhosis and hypoproteinemia. The results showed that Qijia Chaizhu decoction could significantly improve the symptoms of patients with liver cirrhosis and restore serum indicators, such as ALT, TBil, and DBil. Compared with the control group, the curative effect is significant, with an effective rate of 83.33%, which has a significant therapeutic effect on patients with liver cirrhosis and hypoproteinemia.

## 6. Discussion

Currently, modern medicine treatment of hepatitis B liver cirrhosis hypoalbuminemia is mainly to supplement exogenous albumin, amino acids, recombinant human growth hormone, and so forth, thereby enhancing the body's ability to synthesize albumin, and, to a certain extent, it can improve liver function and increase serum albumin levels, prolonging the survival of patients. However, this type of treatment is expensive and requires repeated treatment, which increases patient suffering and is prone to infusion reactions and infectious diseases.

TCM has a unique analysis of the etiology and pathogenesis of LCH. It is believed that insufficient liver blood is the basic pathogenesis, and the insufficiency of water and grain microbes is the main predisposing factor. The symptoms and signs of TCM in patients with LCH include hepatosplenomegaly, jaundice, swollen ascites, dull complexion, pain in the flanks, dark lips and tongue, damp-heat, blood stasis, phlegm and turbidity, as well as visceral *qi* and blood. A number of studies have shown that TCM therapy has a significant protective effect on damaged liver cells in liver cirrhosis. While promoting liver cell repair and regeneration, it can also reduce liver fibrosis, regulate the imbalance of amino acid levels, and form a virtuous cycle, which is beneficial to the synthesis of albumin in the body and has few adverse reactions. Therefore, the long-term efficacy of TCM in the treatment of LCH is more significant, and its related compatibility theory is worth exploring and learning. However, TCM has various types of syndrome differentiation for LCH and different criteria for determining efficacy. In addition, the long-term efficacy observation and evaluation of refractory LCH are less, which leads to certain limitations in the treatment of TCM.

It is well known that the growth hormone (GH)/IGF-Ι axis is important in growth [43, 44]. The presence of binding proteins, particularly IGFBP3, regulates the growth axis by controlling the levels of free IGF-Ι in circulation and prolonging the half-life of IGF-Ι [45]. In LCH patients admitted to hospital due to complications of the disease, IGF-I and IGFBP-3 serum levels were associated with variables related to liver dysfunction and with more advanced liver disease. The levels of these markers mainly reflect hepatic functional status and seem to undergo little influence from other clinical and laboratory variables [46, 47]. Therefore, serum IGF-Ι and IGFBP-3 can provide a new dimension for the evaluation of LCH. This study found that the regulations of IGF-Ι and IGFBP-3 simultaneously exist in a variety of sclerosing hypoproteinemia therapies, suggesting that IGF-Ι and IGFBP-3 are key targets for the treatment of cirrhotic LCH.

At present, modern medical treatment of hypoalbuminemia in liver cirrhosis is mainly to directly supplement exogenous albumin. Other treatment methods include eliminating incentives, increasing synthetic raw materials, and enhancing synthetic functions. These treatments can improve serum albumin levels to a certain extent, but the economic burden of patients is heavy, and the long-term efficacy is not as good as that of traditional Chinese medicine. A number of studies have shown that traditional Chinese medicine has obvious curative effect on the treatment of hypoalbuminemia, and the long-term curative effect is significant, but there are still many problems. ① The research design is not rigorous enough, and the curative effect is mostly limited to dozens of cases. In general, existing studies lack large-scale, multicenter evidence-based studies. ② Differential analysis of hypoalbuminemia in liver cirrhosis is inconsistent for each person, the medication lacks regularity, and there is also a lack of controlled studies between different syndrome differentiation treatments. ③ There is a lack of basic research on Chinese medicine treatment, and the mechanism of Chinese medicine to improve hypoalbuminemia is not clear. It is hoped that research in this area will be strengthened in the future to clarify the mechanism of TCM treatment and to screen out effective prescriptions. In recent years, integrating traditional Chinese and western medicine has had obvious curative effect in the treatment of hypoalbuminemia of liver cirrhosis, and it also shows certain advantages, which can be used as appropriate.

## 7. Conclusion

Hypoalbuminemia has been reported to be closely related to the occurrence and severity of a variety of malignant diseases, such as sepsis, bacteremia, community-acquired pneumonia, and catheter-related bloodstream infection [48]. In the global pandemic of coronavirus disease in 2019 (COVID-19), the liver damage analysis of 2623 hospitalized patients confirmed that hypoproteinemia is a significant risk factor for patients with severe COVID-19 and can independently predict the progression and outcome of COVID-19 [49–51].

At present, hypoproteinemia is still a typical feature of the progression of LCH. It may appear in the early stages of the disease and may remain clinically silent for several years, eventually leading to a variety of complications. In order to improve the quality life of patients and prolong the life cycle, it is particularly important to find accurate and quantified disease severity and safe and effective treatment measures. However, the current clinical trials for the treatment of LCH still have limitations. Many interventions lack rigorous, multicenter, double-blind, and large-sample verification. The prognosis of patients also urgently requires further long-term follow-up trials. There is a lack of systematic medication rules for different syndromes in the treatment of LCH with TCM, and there is a lack of controlled studies between different syndromes. Therefore, in the future, attention should be paid to conducting large-scale, high-quality randomized controlled trials to verify the therapeutic effect of LCH. Simultaneously, researchers should strengthen the systematic study of TCM in the treatment of LCH and clarify the treatment mechanism of TCM, screen core drugs, and form a systematic medication rule to provide reference for clinical treatment of LCH.

## Figures and Tables

**Table 1:** The main prescriptions of the TCM.

Prescription name	Composition	Effects
Fubai formula [33, 36]	Poria, Atractylodis Macrocephalae Rhizoma, Astragali Radix, Angelicae Sinensis Radix, Bubali Cornu, Atractylodis Macrocephalae Rhizoma, Asini Corii Colla, Broussonetiae Fructus	Alleviating the symptoms of patients with liver cirrhosis and hypoalbuminemia, increasing the serum albumin content of patients, and improving the activity of serum cholinesterase in patients
Wuji Baifeng Pills [37]	Wuji, Cervi Cornus Colla, Trionycis Carapax, Ostreae Concha, Mantidis Ootheca, Ginseng Radix et Rhizoma, Astragali Radix, Angelicae Sinensis Radix, Paeoniae Radix Alba, Cyperi Rhizoma, Asparagi Radix, Glycyrrhizae Radix et Rhizoma, Rehmanniae Radix Praeparata, Rehmanniae Radix, Chuanxiong Rhizoma, Stellariae Radix, Salviae Miltiorrhizae Radix et Rhizoma, Dioscoreae Rhizoma, Euryales Semen, Cervi Cornu Degelatinatum	Protecting and improving liver cell function, reducing tyrosine production, promoting tyrosine degradation, and promoting albumin synthesis
Shengbai decoction [39]	Astragali Radix, Drynariae Rhizoma, Codonopsis Radix, Cuscutae Semen, Lycii Fructus, Spatholobi Caulis, Polygoni Multiflori Radix, Angelicae Sinensis Radix, Rehmanniae Radix Praeparata	Increasing the levels of albumin and insulin growth factor-1 in patients with liver cirrhosis and downregulating the serum levels of leptin and growth hormone
Qijia Chaizhu decoction [40, 41]	Astragali Radix, Rhizoma Atractylodis, Gizzard Pepsin, *Polygonum viviparum*, Turtle Shell, *Rehmannia muta*, *Angelica sinensis*, *Salvia miltiorrhiza*	Improving symptoms, signs, liver function, serum albumin, and ultrasonographic findings in patients with liver cirrhosis

## Data Availability

The data used to support the findings of this study are available from the corresponding author upon reasonable request.

## Abbreviations

LCH: Liver cirrhosis with hypoproteinemia
CNKI: China National Knowledge Infrastructure
CBM: Chinese Biomedical Database
VMIS: VIP medicine information system
SBP: Spontaneous peritonitis
GH: Growth hormone
IGF-Ι: Insulin-like growth factor
IGFBP-3: Insulin-like growth factor binding protein-3
HCC: Hepatocellular carcinoma
HBV: Hepatitis B
BCAA: Branched chain amino acids
rhGH: Recombinant human growth hormone
TCM: Traditional Chinese medicine
ChE: Serum cholinesterase
COVID-19: Coronavirus disease 2019.
